# Lack of *KIF21A* mutations in congenital fibrosis of the extraocular muscles type I patients from consanguineous Saudi Arabian families

**Published:** 2011-01-20

**Authors:** Arif O. Khan, Jameela Shinwari, Aisha Omar, Latifa Al-Sharif, Dania S. Khalil, Mohammed Alanazi, Abdullah Al-Amri, Nada Al Tassan

**Affiliations:** 1 Division of Pediatric Ophthalmology, King Khaled Eye Specialist Hospital, Riyadh, Saudi Arabia; 2 Department of Genetics, King Faisal Specialist Hospital & Research Center, Riyadh, Saudi Arabia; 3 Genome Research Unit, Biochemistry Department, King Saud University, Riyadh, Saudi Arabia

## Abstract

**Purpose:**

Congenital fibrosis of the extraocular muscles type I (CFEOM1), the most common CFEOM worldwide, is characterized by bilateral ptotic hypotropia, an inability to supraduct above the horizontal midline, horizontal strabismus (typically exotropia), and ophthalmoplegia with abnormal synkinesis. This distinct non-syndromic phenotype is considered autosomal dominant and is virtually always from heterozygous missense mutations in kinesin family member 21A (*KIF21A*). However, there are occasional *KIF21A*-negative cases, opening the possibility for a recessive cause. The objective of this study is to explore this possibility by assessing CFEOM1 patients exclusively from consanguineous families, who are the most likely to have recessive cause for their phenotype if a recessive cause exists.

**Methods:**

Ophthalmic examination and candidate gene direct sequencing (*KIF21A*, paired-like homeobox 2A [*PHOX2A*], tubulin beta-3 [*TUBB3*]) of CFEOM1 patients from consanguineous families referred for counseling from 2005 to 2010.

**Results:**

All 5 probands had classic CFEOM1 as defined above. Three had siblings with CFEOM. None of the probands had mutations in *KIF21A*, *PHOX2A*, or *TUBB3*.

**Conclusions:**

The lack of *KIF21A* mutations in CFEOM1 patients exclusively from consanguineous families, most of whom had siblings with CFEOM, is strong evidence for a recessive form of CFEOM1. Further studies of such families will hopefully uncover the specific locus(loci).

## Introduction

Congenital fibrosis of the extraocular muscles type 1 (CFEOM1, OMIM 135700) is the most common form of CFEOM reported worldwide [1]. It is a distinct non-syndromic congenital cranial dysinnervation disorder and is characterized by bilateral ptotic hypotropia, an inability to supraduct above the horizontal midline, horizontal strabismus (typically exotropia), and a variable degree of ophthalmoplegia with abnormal synkinesis [2-4]. The phenotype was first mapped as an autosomal dominant fully penetrant trait to the centromere on chromosome 12 [5]. Screening of transcripts in this region in several affected families lead to the discovery of heterozygous missense mutations in kinesin family member 21A (*KIF21A*) as the cause [2]. Since then further studies provide strong evidence that the classic CFEOM1 phenotype results from mutations in *KIF21A* and that sporadic cases are due to de novo mutations in the same gene [1-3,6-14]. A heterozygous *KIF21A* missense mutation has been found to underlie CFEOM1 patients across populations worldwide with most patients having mutations in exon 21 that affect arginine at position 954 of the protein (p.R954W, p.R954Q, or p.R954L) [1-3]. It has been suggested that methylation of CpG dinucleotides in exon 21 of *KIF21A* increases susceptibility to mutational events [13]. Other than exon 21, only 2 other exons in the 38-exon gene have been reported to harbor mutations: exon 8 (p.M356T) and exon 20 (p.E944Q, p.M947V, p.M947T, p.M947R) [3]. The lack of mutations in other exons of *KIF21A* and the lack of other types of mutations (other than missense) may be because such mutations are lethal or because they underlie a phenotype thus far not associated with the gene. The normal function of the KIF21A protein includes the transport of membranous organelles, protein complexes, and mRNAs to specific destinations within the cell in a microtubule- and ATP-dependent manner. These functions are essential for normal morphogenesis and functioning of the cell [15]; however, missense *KIF21A* mutations only appear to significantly affect the orbit, causing widespread orbital dysinnervation [16].

Other clinical forms of CFEOM with a known genetic basis are CFEOM2 (OMIM 602078) and CFEOM3 (OMIM 600638). CFEOM2, the rarest CFEOM phenotype, is a recessive disorder that was first mapped to 11q13 in consanguineous families [17] and later found to be secondary to homozygous mutations in the hindbrain transcription factor paired-like homeobox 2A (*PHOX2A*) [18]. *PHOX2A* knockout animal models reveal that the gene is responsible for development of the oculomotor and trochlear cranial nerve nuclei [19,20]. The CFEOM2 phenotype is characterized by bilateral large-angle exotropia, ptosis, miosis, and ophthalmoplegia with abnormal synkinesis [4,21]. CFEOM3 is CFEOM that does not meet the classic criteria for CFEOM1 or CFEOM2. CFEOM3 can be unilateral or bilateral, is often autosomal dominant, and can have variable penetrance [4,22,23]. A family with autosomal dominant CFEOM is considered to be a CFEOM3 pedigree even if one or more members has (have) classic CFEOM1 if at least one affected family member does not meet the criteria for CFEOM1 [4,23]. Most CFEOM3 families have mapped to 16qter [23-25] and are due to heterozygous mutation in tubulin beta-3 (*TUBB3*), a gene involved in microtubule dynamics, kinesin interactions, and axon guidance [22]. Unlike patients with CFEOM1 or CFEOM2, patients with *TUBB3*-related CFEOM3 can have extraorbital neurologic findings as well [2]. In some instances, CFEOM3 can be caused by heterozygous missense *KIF21A* mutations [23].

CFEOM1 is considered to be an autosomal dominant fully penetrant condition. Although *KIF21A* is the only gene associated with CFEOM1 to date, up to 40% of sporadic CFEOM1 cases do not have identifiable mutations in *KIF21A* [3]. Among the possibly genetic causes are mutations in a *KIF21A* promotor, mutations in *PHOX2A* and/or *TUBB3*, or dominant or recessive mutations at a different locus. If a recessive cause for CFEOM1 exists, one would expect it to occur more commonly in CFEOM1 patients from large consanguineous families [26]. In the current study, we perform candidate gene testing in CFEOM1 patients from consanguineous families to explore the possibility of a recessive cause for the CFEOM1 phenotype.

## Methods

Institutional board approval was granted for this study. Only probands with CFEOM1 who were from consanguineous families were invited to participate in the study. Enrolled patients, who had no known relationship to each other, had complete orthoptic and ophthalmic examination as well as 5 ml venous blood sampling for candidate gene testing and were referred to one of the authors (A.O.K.) from 2005 to 2010. Cyclopentolate 1% was used for dilation and cycloplegic refraction. When affected relatives were available and willing, they were examined as well. The candidate genes *KIF21A*, *PHOX2A*, and *TUBB3* were directly sequenced. Briefly, polymerase chair reaction products from all exons of *KIF21A* (NM_017641), *PHOX2A* (NM_005169), and *TUBB3* (NM_006086.2) were sequenced using the ABI Prism Big Dye Terminator v3.1 Cycle Sequencing Kit as described by the manufacturer.  Results were exported in one of several formats for visualization and sequence was analyzed using SeqMan 6.1 (Lasergene 6 software package) [22,27]. Primers used for *KIF21A* are shown in Table 1, for *PHOX2A* are shown in Table 2, and for *TUBB3* are shown in Table 3 and are as previously published [22].

**Table 1:** Primers for *KIF21A.*

**Exon**	**5′ to 3′ primer sequence**	**PCR conditions**
Kfi21a_x1Fn	ctgttggcttctccacagg	52 °C/35 cycles
Kfi21a_x1Rn	gggactcactgcctcagttt	
Kfi21a_x2Fn	tcatgattttgggggattgt	53 °C/35 cycles
Kfi21a_x2Rn	caaaaatgaaagcgcaactg	
Kif21a_x3F	tcagttgcgctttcatttttg	53 °C/35 cycles
Kif21a_x3R	ctccaacctgggtgacagaa	
Kif21a _x4F	tagcctcattcattttaatgtgtt	59 °C/35 cycles
Kif21a _x4R	gatcttaattccatgtcatgcttc	
Kif21a _x5F	tgcctgtaactgaactaataatgtga	59 °C/35 cycles
Kif21a _x5R	atggctgaccagcttcaact	
Kif21a _x6F	tttggctttatgcctgtttc	59 °C/35 cycles
Kif21a _x6R	tgaggagattggagattcagtg	
Kif21a_x7F	cttatttctgtttcaaagaattagta	59 °C/35 cycles
Kif21a_x7R	cctacacctcaagggatgct	
Kif21a _x8F	caggggcttttaaatttgct	59 °C/35 cycles
Kif21a _x8R	ctccaaaaggaaggaggaca	
Kif21a_x9Fn	tggtcttgaactcctgacctc	59 °C/ 35 cycles
Kif21a_x9rn	tgccctccagaagttaatcc	
Kif21a_x10F	tgtggtctgctcatgtaataaagg	53 °C/35 cycles
Kif21a_x10R	ggaatatgacatcaagggaaagg	
Kif21a_x11Fn	ccacagagaaaaatgctcccta	59 °C/35 cycles
Kif21a_x11Rn	tgaatggaatgcaaaagcag	
Kif21a _x12F	gcatccaagcatgcctaatc	59 °C/35 cycles
Kif21a _x13R	tttaggagcagcccagctta	
Kif21a_x13Fn	tgattggcaatttccattttt	59 °C/35 cycles
Kif21a_x13Rn	gactccccaacacaatgctt	
Kif21a _x14F	gttggggagtcaggggtaga	56 °C/35 cycles
Kif21a _x14R	taaagccttggaaggcaaatg	
Kif21a _x15F	cattcaccttttggttgttgg	59 °C/35 cycles
Kif21a _x15R	aggcacaaactttgacttgc	
Kif21a _x16F	gacaccctagtcttctgagatgtg	59 °C/35 cycles
Kif21a _x16R	ttgccaaaggaaattacatca	
Kif21a _x17F	taaacgtgcagcaaaactgc	59 °C/35 cycles
Kif21a _x17R	tgcttatctattgtccttaacctgc	
Kif21a x18F	tggccgttaatactgaatgttg	56 °C/35cycles
Kif21a x18R	aaagcaggttggattttaagaaa	
Kif21a_x19F	ccatttggaagaaaccttctg	56 °C/35 cycles
Kif21a_x19R	tgcactgccaaataatgagc	
Kif21a _x20–21F	ggcaacaaatggaaacaggt	59 °C/35 cycles
Kif21a _x20–21R	tggcatacatgtaaaacctaagc	
Kif21a _x22F	ccctatgtttcttggggtaatgat	59 °C/35 cycles
Kif21a _x22R	tccttattacaaagcaaagggtta	
Kif21a _x23–24F	ttactggaggagctgggatg	59 °C/35 cycles
Kif21a _x23–24R	tagtgtgtttgtgggcatgg	
Kif21a _x25_ 26F	actaaaaccatcgtgcccat	59 °C/35 cycles
Kif21a _x25_ 26R	gctttagtaaaaccatgccctc	
Kif21a 26F	tggcctagtgaatagcacttagaa	59 °C/35 cycles
Kif21a 26R	cagttaccacttaaagggaaatatga	
Kif21a _x27F	cacacctaggaaaagacacgct	56 °C/35 cycles
Kif21a _x27R	ggggagacaacacctagcaa	
Kif21a_x28F	caagtaataatctttctgaggttcca	56 °C/35 cycles
Kif21a_x28R	accacagcaccagcctaaat	
Kif21a _x29F	ttgttcagaatgcattttatcttaca	59 °C/35 cycles
Kif21a _x29R	gcatggttcctttcccatt	
Kif21a _x30F	agcagggcactatgaaggaa	56 °C/35 cycles
Kif21a _x30R	tttatctaaaaggtatgaccacaaaa	
Kif21a_x31Fn	tgtctcattccctttcacca	56 °C/35 cycles
Kif21a_x31Rn	caacagacttgatctgaaggaga	
Kif21a _x32F	gcttaaaagagagcagtfctgga	59 °C/35 cycles
Kif21a _x32R	ggttgaaccagattatccga	
Kif21a _x33F	tgaagttaggatccttgtggtatg	59 °C/35 cycles
Kif21a _x33R	tgggaagtggacaggtatacaa	
Kif21a _x34F	tgtgttaggtgctgtgctagg	56 °C/35 cycles
Kif21a _x34R	aaggacacaagagacatttagagg	
Kif21a _x35F	gcccaagatcccatctctaa	56 °C/35 cycles
Kif21a _x35R	ccactaactatgaatgaaggaaaaga	
Kif21a_x36Fn	ctccagcctgggaaacatag	59 °C/35 cycles
Kif21a_x36Rn	ggcctgattaatattatctgtaaatga	
Kif21a _x37F	ctttctccagccaattccaa	59 °C/35cycles
Kif21a _x37R	aacctggggtgcctaaattc	
Kif21a _x38F	tgtaaagggcacatggtaacaa	59 °C/35 cycles
Kif21a _x38R	gcagttgaattcagatatattttcca	

**Table 2:** Primers for *PHOX2A*.

**Exon**	**5′ to 3′ primer sequence**	**PCR conditions**
Phox2ax1.1Fn	tccacacctctgagccctaagacgg	63 °C/DMSO10%
Phox2ax1.1Rn	gccgcagggggctgtattggaagc	
Phox2ax1.2fn	ccccgggccgatggactact	63 °C/DMSO10%
Phox2ax1.2Rn	agcgggcccagggattc	
Phox2ax2fn	tcactcccccatcctttttgc	57 °C/35 cycles
Phox2ax2Rn	gctcccacacctccttcca	
Phox2ax3.1fn	gatctcactcgagccttgc	57 °C/35 cycles
Phox2ax3.1Rn	ctgcacgtggactccttgga	
Phox2ax3.2fn	cgggccaagttccgcaaacaggag	57 °C/35 cycles
Phox2ax3.2Rn	ggacgtctctgggggcaggctcgga	

**Table 3:** List of primers for *TUBB3*.

**Exon**	**5’ to 3’ sequence**	**Product size**	**Temperature/cycles**
TUBB3X1F	ggccgcggctataagag	272	56 °C /35
TUBB3X1R	catccctttgttgcaggttc		
TUBB3X2F	tgggtcaaaagccctaatttt	317	56 °C /35
TUBB3X2R	ctgagagctggtgagtccag		
TUBB3X3F	gctcttaggatgtgagcagga	323	56 °C /35
TUBB3X3R	ggagctgaccattccttgtt		
TUBB3X4-1F	atgagaaggggtgctcagtg	489	56 °C /35
TUBB3X4-1R	ctcgttgtcgatgcagtagg		
TUBB3X4-2F	cgcatcatgaacaccttcag	498	56 °C/35
TUBB3X4-2R	gtccacctccttcatggaca		
TUBB3X4-3F	agctcacccagcagatgttc	594	56 °C /35
TUBB3X4-3R	gaggggaaagcagggtgt		

Sequences in Table are based on [22].

## Results

**Figure 1:**
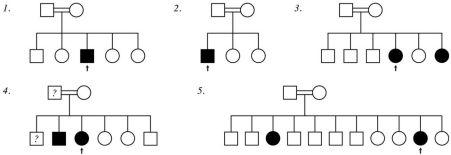
Pedigrees for the five CFEOM probands (arrow indicates proband). All individuals indicated as affected were confirmed to be affected to have CFEOM by examination. Question mark indicates that the individual was described as having strabismus but was not available for confirmatory ophthalmic examination.

**Figure 2:**
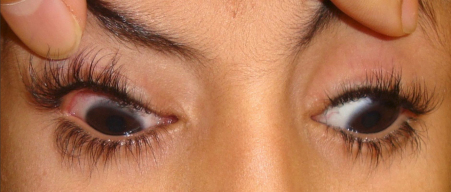
Typical CFEOM1 phenotype. Patient 1 is shown in forced primary position with his eyelids held upward. He has bilateral blepharoptosis, exotropia, hypotropia, and almost complete ophthalmoloplegia. When released, he assumes a chin up position with a left face turn (because of preference for the right eye).

Pedigrees for the 5 patients are shown in Figure 1. All 5 probands had classic CFEOM1 without pupillary abnormality as defined above. One proband had an affected sibling with CFEOM1 (family 3 in Figure 1) and 2 probands each had an affected sibling with CFEOM3 (unilateral CFEOM ; families 4 and 5 in Figure 1). Clinical features of the probands are summarized in Table 4 and the typical proband phenotype is shown in Figure 2 (patient 1 from Table 4). No patient had significant extra-orbital disease.

**Table 4:** Summary of clinical features.

**ID**	**Age**	**Sex**	**Total siblings**	**Family history**	**BCVA**	**Primary**	**AB/AD**	**UP/DN**	**CycloRef**	**Comments**
1	7	M	5	none	20/20	xt/hypo	−4/-2	−6/-1	+0.50	attempt up=ad
					20/30	xt/hypo	−4/-2	−6/-1	+0.50	attempt up=ad
2	7	M	3	maternal uncle with bilateral ptosis	20/40	xt/hypo	−4/-2	−6/-1	+2.00–2.75x015	attempt up=ad
					20/400	xt/hypo	−4/-2	−6/-1	−5.00–2.00x150	attempt up=ad
3	9	F	6	Younger sister with CFEOM1	20/70	xt/hypo	−1/-1	−5/0	+2.50–2.00x180	attempt ad=dn; attempt up=ad
					20/60	xt/hypo	−1/-1	−5/0	+3.50–3.25x180	attempt ad=dn; attempt up=ad
4	13	F	6	Older brother with CFEOM3 in left eye	20/30	et/hypo	−2/0	−5/0	+2.00	attempt ab=dn; attempt up=ad; torsional nystagmus
					20/25	et/hypo	−3/0	−5/0	+2.00	attempt up=ad; torsional nystagmus
5	17	F	11	Older sister with CFEOM3 in left eye	20/40	xt/hypo	−2/-3	−5/-3	+2.00	attempt up=dn&ad
					20/60	xt/hypo	−2/-1	−5/-3	+8.00	attempt up=ab

For each patient where relevant, first row represents right eye data and second row represents left eye data; AGE:age in years; F:female; M:male; BCVA:best-corrected visual acuity; CSM:central steady maintained; et:esotropia; xt:exotropia; hypo: hypotropia;ab/ad:limitation of abduction/adduction on a scale of 0 to −4 (−5=eye cannot reach primary); CycloRef:cycloplegic refraction; up/down:limitation of supraduction/infraduction on a scale of 0 to −4 (−5=eye cannot reach primary); attempted x=y: when x attempted, y inappropriately occurs (dysinnervation).

No proband had mutations or polymorphic variations in *KIF21A*, *PHOX2A*, or *TUBB3*.

## Discussion

Five CFEOM1 probands from consanguineous families were assessed in this study, none of whom had significant extra-orbital disease. Two were sporadic cases, one had a sibling with CFEOM1, and 2 each had a sibling with CFEOM3. No proband from this unique CFEOM1 cohort harbored mutations in *KIF21A*, *TUBB3*, or *PHOX2A*. Rather than being an autosomal dominant phenotype, CFEOM1 in our cohort was almost certainly related to homozygous mutations in a locus (or in loci) that to date has (or have) not been associated with the condition.

The 3 previously-reported CFEOM1 families from Saudi Arabia were not consanguineous and all harbored heterozygous missense *KIF21A* mutations [6,9]. Two families had autosomal dominant inheritance and both harbored the most common *KIF21A* mutation reported worldwide, p.R954W [9]. The third family, of Jordanian ancestry, exhibited apparent autosomal recessive inheritance with atypical abnormal pupils but in fact harbored heterozygous p.R954L KIF21A mutation with parental germline mosaicism [6]. In the current series, none of the 5 CFEOM1 patients harbored mutations in known CFEOM genes. Two cases were sporadic and 3 had affected siblings. For one, the sibling also had CFEOM1 (family 3 from Figure 1). For the other 2, the each had an affected sibling with CFEOM3 (families 4 and 5 from Figure 1). These latter 2 families would be considered by some authors as CFEOM3 pedigrees [4,23].

Studies of consanguineous families are more likely to uncover recessive cause for a given phenotype if a recessive cause exists because of parental shared recent ancestry. Although every individual is a heterozygous carrier for mutated alleles that would potentially cause recessive disease in the homozygous (or compound heterozygous) state, it is unlikely that the individual's spouse will carry the same disorder unless they are related [28]. Thus studies of exclusively consanguineous families with a specific phenotype offer a unique opportunity to uncover a recessive cause for the phenotype if a recessive cause exists. Our study confirms the existence of recessive CFEOM1. There may be one or more such loci, each of which may be a separate gene or locus that regulates pathways in known genes associated with CFEOM. Whether the 2 families that included a sibling with CFEOM3 (families 4 and 5 from Figure 1) are considered CFEOM3 families or families with CFEOM1 probands, the observed phenotype is likely related to a recessive cause that has not yet been described.

In summary, although most CFEOM1 is due to heterozygous missense *KIF21A* mutations, there exists at least one additional autosomal recessive cause for the phenotype. This information is useful in the genetic counseling of sporadic *KIF21A*-negative CFEOM1 patients. It is hoped that further ascertainment and study of CFEOM1 patients from consanguineous families will uncover the novel locus(loci).
